# The presence of anti-nuclear antibodies alone is associated with changes in B cell activation and T follicular helper cells similar to those in systemic autoimmune rheumatic disease

**DOI:** 10.1186/s13075-018-1752-3

**Published:** 2018-11-29

**Authors:** Yuriy Baglaenko, Nan-Hua Chang, Sindhu R. Johnson, Waleed Hafiz, Kieran Manion, Dario Ferri, Babak Noamani, Dennisse Bonilla, Sina Rusta-Sellehy, Larissa Lisnevskaia, Earl Silverman, Arthur Bookman, Carolina Landolt-Marticorena, Joan Wither

**Affiliations:** 1 Krembil Research Institute, University Health Network, 60 Leonard Avenue, 5KD402, Toronto, ON M5T 2S8 Canada; 2 Department of Immunology, University of Toronto, Toronto, ON Canada; 3 Division of Rheumatology, University Health Network, Toronto, ON Canada; 4 Department of Medicine, University of Toronto, Toronto, ON Canada; 5 Division of Rheumatology, Mount Sinai Hospital, Toronto, ON Canada; 6 Institute of Health Policy, Management and Evaluation, University of Toronto, Toronto, ON Canada; 7 Lakeridge Health Services, Oshawa, ON Canada; 8 Division of Rheumatology, Sick Children’s Hospital, Toronto, ON Canada; 9 Department of Pediatrics, University of Toronto, Toronto, ON Canada

**Keywords:** Systemic autoimmune rheumatic disease, Anti-nuclear antibodies, B cell, T cell

## Abstract

**Background:**

Diagnosis of systemic autoimmune rheumatic diseases (SARD) relies on the presence of hallmark anti-nuclear antibodies (ANA), many of which can be detected years before clinical manifestations. However, ANAs are also seen in healthy individuals, most of whom will not develop SARD. Here, we examined a unique cohort of asymptomatic ANA^+^ individuals to determine whether they share any of the cellular immunologic features seen in SARD.

**Methods:**

Healthy ANA^−^ controls and ANA^+^ (ANA ≥1:160 by immunofluorescence) participants with no SARD criteria, with at least one criterion (undifferentiated connective tissue disease (UCTD)), or meeting SARD classification criteria were recruited. Peripheral blood cellular immunological changes were assessed by flow cytometry and transcript levels of *BAFF*, interferon (IFN)-induced and plasma cell-expressed genes were quantified by NanoString.

**Results:**

A number of the immunologic abnormalities seen in SARD, including changes in peripheral B (switched memory) and T (iNKT, T regulatory, activated memory T follicular helper) subsets and B cell activation, were also seen in asymptomatic ANA^+^ subjects and those with UCTD. The extent of these immunologic changes correlated with ANA titer or the number of different specific ANAs produced. Principal component analysis of the cellular data indicated that a significant proportion of asymptomatic ANA^+^ subjects and subjects with UCTD clustered  with patients with early SARD, rather than ANA^−^ healthy controls.

**Conclusions:**

ANA production is associated with altered T and B cell activation even in asymptomatic individuals. Some of the currently accepted cellular features of SARD may be associated with ANA production rather than the immunologic events that cause symptoms in SARD.

**Electronic supplementary material:**

The online version of this article (10.1186/s13075-018-1752-3) contains supplementary material, which is available to authorized users.

## Background

Within the group of systemic autoimmune rheumatic diseases (SARD), systemic lupus erythematosus (SLE), Sjogren’s disease (SjD), systemic sclerosis (SSc), dermatomyositis, and mixed connective tissue disease appear to share a similar pathogenesis based upon their production of anti-nuclear antibodies (ANAs), overlapping clinical features, co-segregation within families, and shared genetic risk variants [1–4]. Studies of patients with SLE and SjD prior to diagnosis indicate a prolonged pre-clinical phase during which ANAs can be detected in the absence of clinical symptoms [5–8]. While this observation suggests that ANA positivity might serve as a biomarker for SARD development, ANAs are also seen in the healthy population and based upon their prevalence together with that of SARD, it is estimated that > 90% of ANA^+^ individuals will not progress to SARD.

Following a variable period of asymptomatic ANA positivity, individuals who progress to SLE have the insidious onset of accumulating clinical symptoms, culminating in sufficient criteria for diagnosis [5, 6]. It is likely that the other SARD have a similar course, since it is not uncommon for individuals to present with positive serologic findings and some clinical symptoms of SARD but insufficient symptoms/signs to make a definitive diagnosis [9–17]. Approximately 20–40% of these patients go on to develop SARD over the next 3–5 years [11, 13–15]. Although various serologic and cytokine profiles have been reported to be associated with an increased risk of SARD progression [18–22], the cellular immune changes that accompany these serologic/cytokine changes and that distinguish individuals that will eventually progress to SARD from those who will not remain to be determined. In this study, we examined peripheral blood T and B cell populations and their activation in asymptomatic ANA^+^ individuals together with patients with undifferentiated connective tissue disease (UCTD) and early SARD to determine whether the cellular immune characteristics found in SARD are distinct from those seen in asymptomatic ANA^+^ individuals, most of whom will not progress to SARD. Surprisingly, several of the cellular changes seen in SARD are also seen in asymptomatic ANA^+^ individuals, suggesting that they are associated with ANA production rather than development of symptoms in SARD.

## Methods

### Subjects and data collection

ANA^+^ subjects were recruited at the Toronto Western and Mount Sinai Hospitals. Patients were typically referred to clinic because of a recently discovered positive ANA test with or without rheumatologic symptoms. All patients were assessed by a participating rheumatologist and the clinical data recorded on a standardized data retrieval form. Individuals with ANA ≥1:160 were stratified into 3 groups based upon the presence of SARD clinical diagnostic criteria (1997 American College of Rheumatology (ACR) criteria for SLE [23], 2013 ACR-European League Against Rheumatism (EULAR) criteria for SSc [24], or the revised American-European criteria for SjD [25]): (1) asymptomatic ANA^+^, no clinical criteria of SARD; (2) UCTD, ≥ 1 clinical symptom of SARD but insufficient criteria for diagnosis; or (3) early SARD, meeting classification criteria and within 2 years of diagnosis. None of the subjects were on any corticosteroids or disease-modifying antirheumatic drugs (DMARDs), with the exception of anti-malarials. Sex-matched healthy controls (HC) were recruited from hospital/laboratory personnel and were ANA and specific anti-nuclear antibody negative. Information on family history of SARD or rheumatoid arthritis was ascertained using a validated questionnaire [26]. The study was approved by the Research Ethics Boards of both recruiting hospitals and all participants gave signed informed consent.

### Cellular characterization

Peripheral blood mononuclear cells (PBMCs) were isolated over a Ficoll/Hypaque (GE Healthcare) gradient and treated to remove residual red blood cells (RBCs). Freshly isolated cells (0.5 × 10^6^), or for T cell intracellular-cytokine expression 1 × 10^6^ cells stimulated with 50 ng/mL phorbol-12-myristate-13-acetate (PMA) and 500 ng/mL ionomycin for 4–5 h in the presence of GolgiStop (BD Biosciences), were stained with various combinations of directly conjugated monoclonal antibodies (mAbs). Antibodies used for staining were mouse anti-human FOXP3-PE (259D/C7), invariant NKT cell-PE (6B11), CD45RA-PECy7 (HI100), CD3-APC H7 (SK7), CD4-Pacific Blue (RPA-T4), and CD8-PerCPCy5.5 (RPA-T8) from BD Biosciences; and mouse anti-human CD21-FITC (BU32), CD95-FITC (DX2), CXCR5-AlexaFluor488 (J252D4), HELIOS-FITC (22F6), CD24-PE (ML5), CD138-PE (DL-101), IgD-PerCPCy5.5 (IA6–2), CD38-PECy7 (HB-7), IgM–APC (MHM-88), CD25-APC (M-A251), CD27-APC/Cy7, CD19-brilliant Violet 421 (HIB19), and CD86-brilliant Violet 605 (IT2.2) from BioLegend. Staining for intracellular FOXP3 and HELIOS was performed according to the protocol from BD Biosciences, using nuclear Cytofix/Cytoperm solutions. For assessment of T cell intracellular cytokine expression, stimulated cells were first stained for cell surface markers (anti-CD3, anti-CD4, and anti-CD8). The cells were then fixed for 30 min on ice with Cytofix/Cytoperm, washed, and stained in Cytoperm with anti-IL-17A-PE (ebio64CAP17, ThermoFisher Scientific) and -IL-21-APC (3A3-N2, BD BioSciences), or anti-IFNγ-APC (4S.B3, ThermoFisher Scientific) for 30 min on ice. Following further washing, events were acquired using a three-laser LSRII or FACSCanto (BD Biosciences) flow cytometer, with fluorescence-minus-one controls used as negative staining controls. Cells were gated and analyzed using Flow Jo software (TreeStar).

### Cytokine measurement

Total RNA was isolated from whole peripheral blood archived in Tempus tubes (Applied Biosystems) and gene expression was quantified by NanoString using a custom array (nanoString Technologies), as previously described [27]. Log_2_ normalized expression levels of 5 interferon (IFN)-induced genes (*EPSTI1*, *IFI44L*, *LY6E*, *OAS3*, *RSAD2)* and 5 plasma cell (PC)-expressed genes (*IGHA1, IGJ, IGKV401, IGKC, TNFRSF17*) were summed to generate IFN5 and PC5 scores, respectively. Serum IFN-α and B cell activating factor (BAFF) levels were quantified by ELISA, as previously described [27].

### Measurement of autoantibodies

ANAs were quantified by indirect immunofluorescence using the Kallestad® Hep-2 kit (BioRad), through the University Health Network laboratory. The Bioplex® 2200 ANA Screening System (BioRad) was used to measure the serum levels of 11 specific autoantibodies (anti-dsDNA, anti-chromatin, anti-Ro, anti-La, anti-Sm, anti-SmRNP, anti-RNP, anti-Jo-1, anti-Scl-70, anti-centromere and anti-ribosomal P), using the company’s cutoffs. HC with ANA ≥1:160 were re-classified into the asymptomatic ANA^+^ group and those with positive ANA <1:160 or any specific autoantibodies were excluded from the study.

### Data analysis

The Kruskal-Wallis test was used for statistical comparisons of differences between ≥ 3 groups, followed by Dunn’s post-test for multiple comparisons. The Mann-Whitney *U* test was performed to compare continuous variables between two groups and Fisher’s exact test was used to compare discrete variables. The strength of association between variables was determined using Spearman’s correlation coefficient. All statistical analyses were performed using GraphPad 6 software (La Jolla, CA, USA) or using various packages in R. Correlation matrices were created using the corrplot (v0.84) package. Principal component analyses (PCA) were performed using the PCA function in the missMDA (v1.12) package, with missing data imputed using the imputePCA function. A total of 10 PCs were calculated. Corresponding plots were created using the scatterplot3d (v0.3–41) package.

## Results

### ANA^+^ individuals lacking a SARD diagnosis have an altered immunologic phenotype

Demographic and relevant clinical/serologic information for the 187 study participants is shown in Table 1 and (see Additional file 1: Table S1). ANA testing in ANA^+^ individuals lacking SARD criteria was performed for a variety of reasons including: non-inflammatory arthritis/arthralgias (41%, mostly osteoarthritis and fibromyalgia), recruitment to the study as a healthy control (18%), healthy mother with recurrent miscarriage or child with neonatal lupus (13%), family history of autoimmunity (7%), urticaria/non-specific rash (7%), sicca symptoms in the absence of objective signs of dryness (5%), fatigue (3%), or other (7%). ANA^−^ HCs were significantly younger than any of the ANA^+^ groups and a larger proportion of the group was non-Caucasian than in the UCTD and SARD groups (see Additional file 1: Table S1 for additional ethnicity information). There were no significant differences between groups in the proportion of subjects taking anti-malarials. A small number (*n* = 5) of the asymptomatic ANA^+^ individuals were taking anti-malarials at the time of initial evaluation in clinic, which had been started for vague symptoms (fatigue, fibromyalgia) that could not be definitively attributed to SARD. Patients with early SARD had significantly higher ANA titers and a larger number of nuclear antigen autoantibody specificities (as determined by the Bioplex®) when compared with asymptomatic ANA^+^ subjects and subjects with UCTD (Table 1). Additional details on the number and types of ANAs seen in each of the different ANA^+^ groups can be found in Additional file 1: Table S1.

**Table 1:** Study participant characteristics

	HC	Asymptomatic	UCTD	SARD				
	ANA^−^ *N* = 32	ANA^+^ *N* = 61	*N* = 35	Total *N* = 59	SSc *N* = 19	SLE *N* = 10	SjD *N* = 28	DM/MCTD *N* = 2
Sex: *n* Female (%)	29 (91)	59 (97)	33 (94)	55 (93)	17 (89)	10 (100)	26 (93)	2 (100)
Age: mean ± SD	35.1 ± 11.8	**44.1 ± 13.9** ^**a**^	**46.5 ± 16.3**	**50.7 ± 13.7**	55.1 ± 12.9	37.3 ± 10.9	53.0 ± 12.3	44
Anti-malarials: *n* (%)	0 (0)	5 (8.2)	8 (22.8)	5 (8.5)	1 (5.3)	2 (20)	2 (7.1)	0 (0)
Ethnicity: *n* Caucasian (%)	12 (37.5)	36 (59.0)	**24 (68.6)**	**39 (66.1)**	13 (68.4)	5 (50)	20 (71.4)	1 (50)
Family history: *n* (%)^b^	1 (3.1)	**15 (25.9)**	**7 (21.9)**	**15 (26.8)**	4 (23.5)	1 (11.1)	9 (31.2)	1 (50)
ANA titer: median	N/A	1/640^c^	1/640^c^	> 1/640	> 1/640	> 1/640	1/640	> 1/640
Number of Abs: Mean ± SD	N/A	0.74 ± 1.05^c^	0.94 ± 1.17^c^	1.92 ± 1.32	1.32 ± 0.80	2.7 ± 2.45	2.04 ± 0.63	2.5

*Abbreviations: HC* healthy control, *ANA* anti-nuclear antibody, *UCTD* undifferentiated connective tissue disease, *SARD* systemic autoimmune rheumatic disease, *SSc* systemic sclerosis, *SLE* systemic lupus erythematosus, *SjD* Sjogren’s disease, *DM/MCTD* dermatomyositis or mixed connective tissue disease, *N* number, *SD* standard deviation, *Abs* antibodies

^a^Values significantly (*p* < 0.05) different from ANA^−^ HC are in bold

^b^Family history of SARD or rheumatoid arthritis. Percentages are presented as a proportion of those whose family history is known

^c^Significantly (*p* < 0.05) different from SARD

**Fig. 1:**
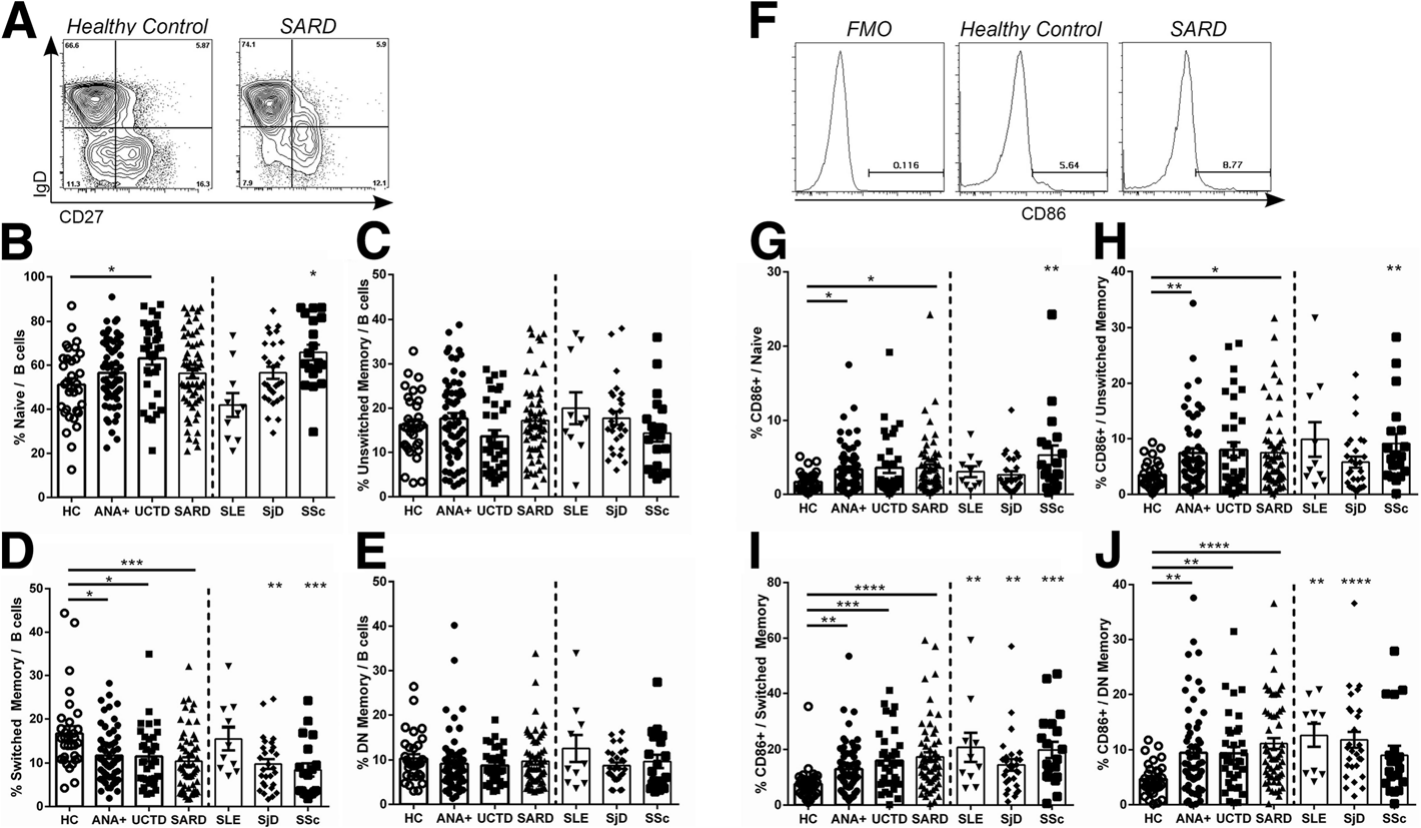
Asymptomatic anti-nuclear antibodies (ANA)^+^ individuals with no diagnosis of systemic autoimmune rheumatic disease (SARD) have abnormalities in peripheral B cell subsets and their activation similar to symptomatic patients with SARD. **a** Gating strategy for identification of naïve (IgD^+^CD27^−^), unswitched memory (IgD^+^CD27^+^), switched memory (IgD^−^CD27^+^), and double-negative (DN) memory (IgD^−^CD27^−^) cells within the CD19^+^ B cell compartment of peripheral blood mononuclear cells from a representative healthy control and a patient with SARD. **b–e** Proportions of the different peripheral B cell subsets within the CD19^+^ B cell compartment for each subject group. **f** Representative gating for CD86^+^ cells within the various B cell compartments (example shown is gated on all CD19^+^ B cells). **g–j** Proportion of CD86^+^ cells within each of the different peripheral B cell subsets. Statistical comparisons on the left side of each plot are between healthy controls (HC) and asymptomatic ANA^+^ individuals (ANA+), patients with undifferentiated connective tissue disease (UCTD), or pooled patients with SARD, whereas those on the right side of the plot are comparisons between the individual SARDs and HC. Bars represent the mean with SEM. Every data point represents an individual patient. For each set of comparisons statistical significance was determined using the Kruskal-Wallis test with Dunn’s post-hoc test for multiple comparisons, as compared to HC; *p ≤ 0.05, **p ≤ 0.01, ***p ≤ 0.001, ****p ≤ 0.0001. SLE, systemic lupus erythematosus; SjD, Sjogren’s disease; SSc, systemic sclerosis

To determine whether ANA^+^ individuals not diagnosed with SARD share any of the immunologic changes seen in SARD, several peripheral blood immune T and B cell populations were examined using flow cytometry. Similar to SARD, the number of PBMCs and lymphocytes per milliliter of blood were significantly decreased in asymptomatic ANA^+^ subjects and subjects with UCTD as compared to ANA^−^ HC (all *p* < 0.05). Previous work has shown changes in the proportion and activation of various B cell subsets in SARD [28–37]. Therefore, the proportions of naïve (IgD^+^CD27^−^), unswitched memory (IgD^+^CD27^+^), switched memory (IgD^−^CD27^+^), and double-negative memory (IgD^−^CD27^−^) cells within the CD19^+^ B cell population were assessed and the percentage of activated cells within each B cell subset quantified by staining with anti-CD86 or anti-CD95. As previously reported [28], the proportion of naïve B cells was increased in patients with SSc as compared to ANA^−^ HC. This was not seen when the SARD population was examined as a whole, reflecting the lack of significant changes in the SLE and SjD patient subsets. With the exception of a reduction of switched memory B cells, the proportions of the remaining B cell subsets did not differ between ANA^−^ HC and any of the individual SARDs or the SARD population as a whole. In general, the changes seen in the asymptomatic ANA^+^ subjects and subjects with UCTD (Fig. 1a–e) paralleled those seen in patients with early SARD, with both groups demonstrating significant decreases in the proportion of switched memory B cells and the number of these cells/milliliter of blood (both *p* < 0.01). There were no differences in the absolute number of cells in the blood for any of the other B cell populations examined, including the transitional B cell population.

All ANA^+^ individuals, regardless of disease state, had increased expression of the activation marker CD86 on all of their B cell sub-populations. Although there was some variation between the individual SARD conditions in the extent of B cell activation seen in the different B cell subsets, overall the most significant differences as compared to ANA^−^ HC were seen in the switched memory and double-negative memory B cell compartments, and this was recapitulated in the asymptomatic ANA^+^ subjects and subjects with UCTD (Fig. 1f–j). Similar trends were seen for CD95 expression, but in general were not statistically significant (see Additional file 1: Figure S1).

Despite increases in global B cell activation, the frequencies of PC and/or plasmablasts were only significantly elevated in patients with early SLE as compared to ANA^−^ HC (see Additional file 1: Figure S2), consistent with previous reports [38]. There was considerable variability in the levels of these cells between individuals within the asymptomatic ANA^+^ and UCTD groups, with a non-significant trend toward increased proportions of plasma cells and/or plasmablasts as compared to HC. This variability did not appear to be due to a lack of consistency in the gating of these populations, as there was a strong correlation between the proportion of PC and/or plasmablasts measured by flow cytometry and genes comprising the PC5 score (see Additional file 1: Figure S2), as reported in other studies [39].

**Fig. 2:**
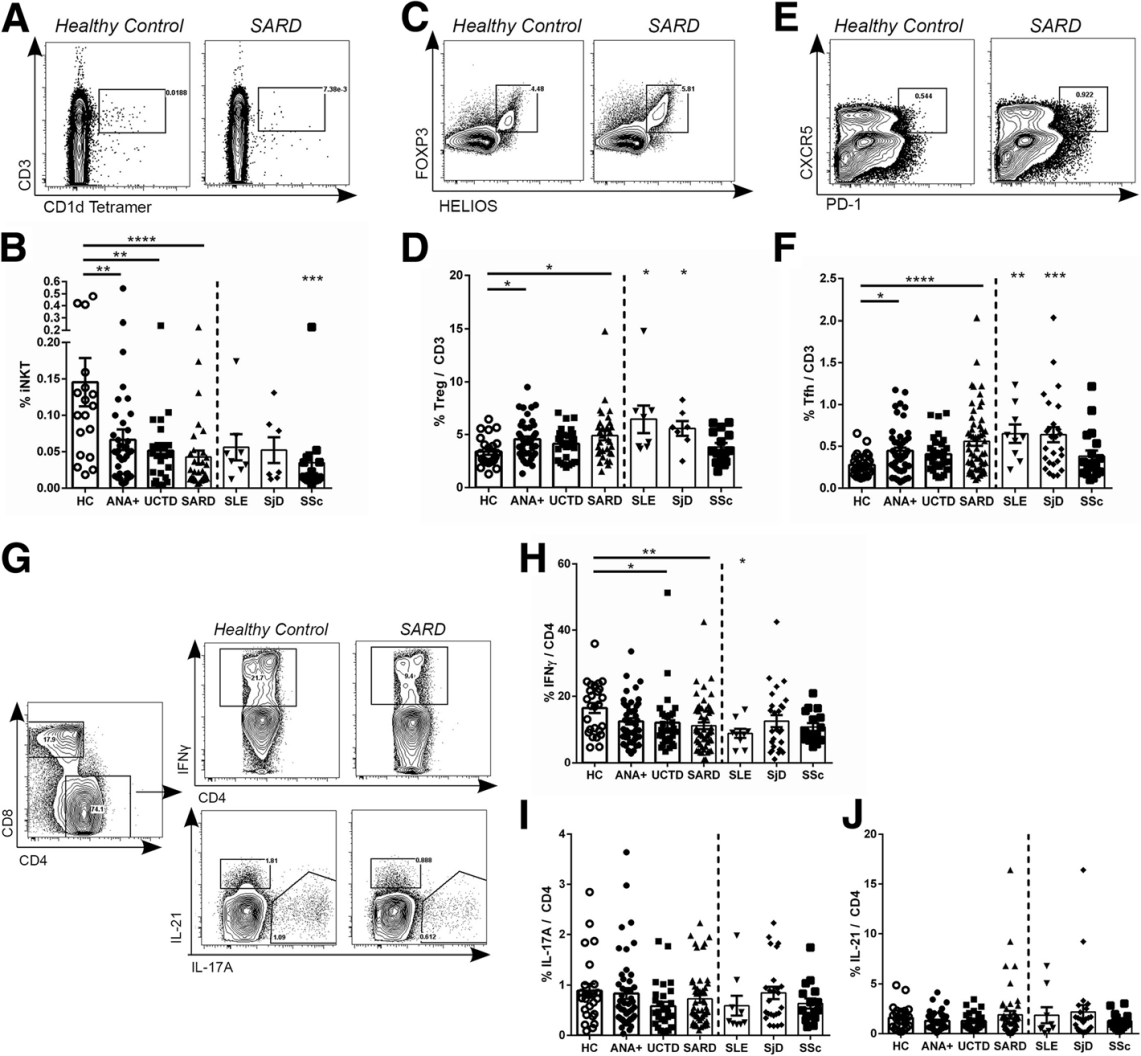
Reductions in invariant natural killer T (iNKT) cells and increases in T follicular helper (Tfh) and T regulatory (Treg) cells in anti-nuclear antibodies (ANA)^+^ individuals regardless of diagnosis. Representative gating, together with scatterplots show frequencies in each subject group for peripheral iNKT (**a**, **b**), Treg (**c**, **d**), and Tfh (**e**, **f**) cells. For the iNKT cell plot, the cells were first gated on lymphocytes, whereas for the Tfh and Treg cell plots, they were first gated on CD3^+^CD4^+^ cells within the lymphocyte pool with the scatterplots showing the percentage of these cells as a proportion of the CD3^+^ T cell population. **g** Representative gating for interferon (IFN)-γ-, IL-17A-, and IL-21-producing CD4 T cells following phorbol-12-myristate-13-acetate (PMA) and ionomycin stimulation of peripheral blood mononuclear cells (plots shown have been gated on CD3^+^CD4^+^ lymphocytes). **h–j** Proportions of cytokine-producing cells within the CD3^+^CD4^+^ T cell population for each subject group. Statistical comparisons on the left side of each figure are between healthy controls (HC) and asymptomatic ANA^+^ individuals (ANA+), patients with undifferentiated connective tissue disease (UCTD), or pooled patients with systemic autoimmune rheumatic disease (SARD), whereas those on the right side of the figure are between the individual SARDs and HC. Bars represent the mean with SEM. Every data point represents an individual patient. Statistical significance was determined using the Kruskal-Wallis test with Dunn’s post-hoc test for multiple comparisons, as compared to HC; *p ≤ 0.05, **p ≤ 0.01, ***p ≤ 0.001, ****p ≤ 0.0001. SLE, systemic lupus erythematosus; SjD, Sjogren’s disease; SSc, systemic sclerosis

Given the similarity in the B cell phenotype between ANA^+^ subjects with and without a SARD diagnosis, we questioned whether alterations in T cell regulation/activation were also shared. As previously reported, peripheral iNKT cell frequencies are greatly reduced in the various SARD conditions [40–44]. Similar reductions were seen in the patients with early SARD examined here, and these findings were recapitulated in asymptomatic ANA^+^ subjects and subjects with UCTD (Fig. 2a, b).

Although it was originally proposed that the frequency of T regulatory (Treg) cells is deceased in SARD, several recent papers have provided data indicating that the proportion of Treg cells is actually increased in SLE and SjD [45–48]. Consistent with these findings, we observed an increased frequency of HELIOS^+^FOXP3^+^ Treg cells in SLE and SjD and in the early SARD group as a whole, as compared to ANA^−^ HC. Again, similar trends were seen in ANA^+^ individuals with no SARD diagnosis to those seen in SARD, which was statistically significant in the asymptomatic ANA^+^ group (Fig. 2c, d).

To investigate T cell activation, we assessed the proportion of PD-1^hi^CXCR5^+^ T follicular helper cells (Tfh, Fig. 2e, f), which has been previously shown to reflect activated memory Tfh cells [49], and the proportions of cytokine-producing T cells (Fig. 2g–j). Consistent with previous reports of increased activated memory Tfh cells in SLE and SjD [34, 50, 51], the proportion of these cells was significantly increased in both these conditions and in the early SARD group as a whole. Similar but slightly less significant changes were seen in asymptomatic ANA^+^ subjects and subjects with UCTD.

There is considerable disagreement between studies with regard to whether the proportions of various cytokine-producing cells within the CD4^+^ T cell population in the SARD conditions differ from those of healthy controls and if so, whether they are increased or decreased [52–61]. Despite a report indicating that the proportion of peripheral blood IFN-γ-producing CD4^+^ cells in patients with UCTD and SARD are increased as compared to HC [54], we found that the proportion of these cells was reduced in patients with early SARD, as reported by some groups [56, 58, 61], and that a similar trend was seen for the ANA^+^ subjects with no SARD diagnosis, and this was statistically significant in patients with UCTD. The reduced levels of these cells were not accompanied by changes in serum IFN-γ (data not shown). No significant differences were seen in the proportions of IL-17-producing and IL-21-producing cells within the peripheral blood in any subject group as compared to ANA^−^ HC. When the number of cells/milliliter of blood was calculated for each T cell population, only the reduced proportion of iNKT cells was significant (*p* < 0.01 for all 3 ANA^+^ groups).

Although we endeavored to age-match, sex-match, and ethnicity-match our ANA^−^ HC with the various ANA^+^ groups, some differences remained. To address whether these differences might have contributed to the differences observed between the ANA^+^ and ANA^−^ groups, we examined the association between these demographic parameters and the cellular phenotypes that were statistically significant. As shown in (see Additional file 1: Figure S3), the majority of the cellular phenotypes did not vary with age. The only exceptions were the percentage of CD86^+^ naïve B cells in patients with SARD, which positively correlated with age (*r* = 0.28, *p* = 0.03) and the proportion of iNKT cells, which negatively correlated with age in ANA^−^ HC (*r* = − 0.56, *p* = 0.02) and patients with SARD (*r* = − 0.51, *p* = 0.003). The cellular phenotypes also did not vary with ethnicity in ANA^−^ HC. In asymptomatic ANA^+^ individuals, the proportions of Tfh (*p* = 0.02) and iNKT cells (*p* = 0.03) were slightly increased in non-Caucasians as compared to Caucasians, while in patients with SARD the proportion of CD86^+^ cells in the naïve B cell compartment was decreased in non-Caucasians (*p* = 0.02). With the possible exception of iNKT cells, these trends could not account for the differences observed between groups.

### The autoantibody profile correlates independently with the levels of type I IFN and Tfh cells

As ANA^+^ individuals with no SARD diagnosis had a range of ANA titers and number/type of specific anti-nuclear antibodies, a Spearman correlation matrix was produced to investigate the association between serologic changes and the peripheral blood cellular profile. BAFF and type I IFN, as measured by peripheral blood gene expression or serum levels, was also included in this analysis, as these cytokines have been proposed to promote immune dysregulation in SARD [62, 63] and we have previously shown that they are elevated in a subset of ANA^+^ individuals with no SARD diagnosis ([27] and Additional file 1: Figure S4).

**Fig. 3:**
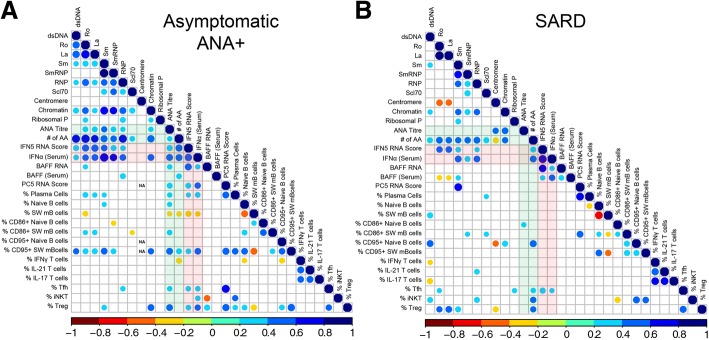
Correlation between cellular and selected serologic/cytokine phenotypes in asymptomatic anti-nuclear antibodies (ANA)^+^ individuals and patients with systemic autoimmune rheumatic disease (SARD). Spearman correlation matrices for asymptomatic ANA^+^ individuals (**a**) and patients with SARD (**b**). B and T cell populations were gated and defined as outlined in Figs. 1 and 2, plasma cells as outlined in Additional file 1: Figure S3, and CD95^+^ B cell subpopulations as shown in Additional file 1: Figure S1. The color and size of the dots represents the *ρ* value, with the scales shown at the bottom of each matrix. Non-significant (*p* ≥ 0.05) correlation is not displayed. IFN, interferon; BAFF, B cell activating factor

In asymptomatic ANA^+^ individuals, the ANA titer was negatively correlated with the proportion of switched memory B cells, and positively correlated with markers of activation (CD86 and CD95) in this B cell compartment, and with the proportion of plasma cells, activated memory Tfh, Treg cells, and the levels of type I IFN/BAFF (Fig. 3). Similar associations were seen for the number of different autoantibody specificities detected by BioPlex®, except that there was a negative correlation with the proportion of IFN-γ-producing T cells and no correlation with plasma, Tfh, or Treg cells. In patients with early SARD (Fig. 3), no cellular associations were seen with ANA titer. However, patients with early SARD demonstrated the same positive association between the number of autoantibody specificities and the percentage of CD95^+^ switched memory B cells, proportions of Tfh and Treg cells, and levels of type I IFN/BAFF as was seen in ANA^+^ asymptomatic individuals with ANA titer, arguing that the same immunologic processes are involved in the generation of autoantibodies in symptomatic and asymptomatic individuals. Overall similar but less significant findings were observed in patients with UCTD (see Additional file 1: Figure S5), most likely due to the smaller number of these patients in the cohort.

Since many of the cellular phenotypes appeared to correlate with each other, multivariate analysis was performed to determine which phenotypes were independent predictors of serologic status. When all ANA^+^ subjects were included (those with asymptomatic ANA^+^, UCTD, and SARD), a model including IFN5 RNA score (β = 0.0519, *p* = 2.62e-13), serum IFN-α levels (β = 0.0646, *p* = 1.43e-07) and the frequency of Tfh cells (β = 0.152, *p* = 0.0279) best predicted the number of autoantibody specificities. These findings suggest that type I IFNs and Tfh cells independently drive the immune dysregulation that leads to production of multiple autoantibodies in ANA^+^ individuals.

**Fig. 4:**
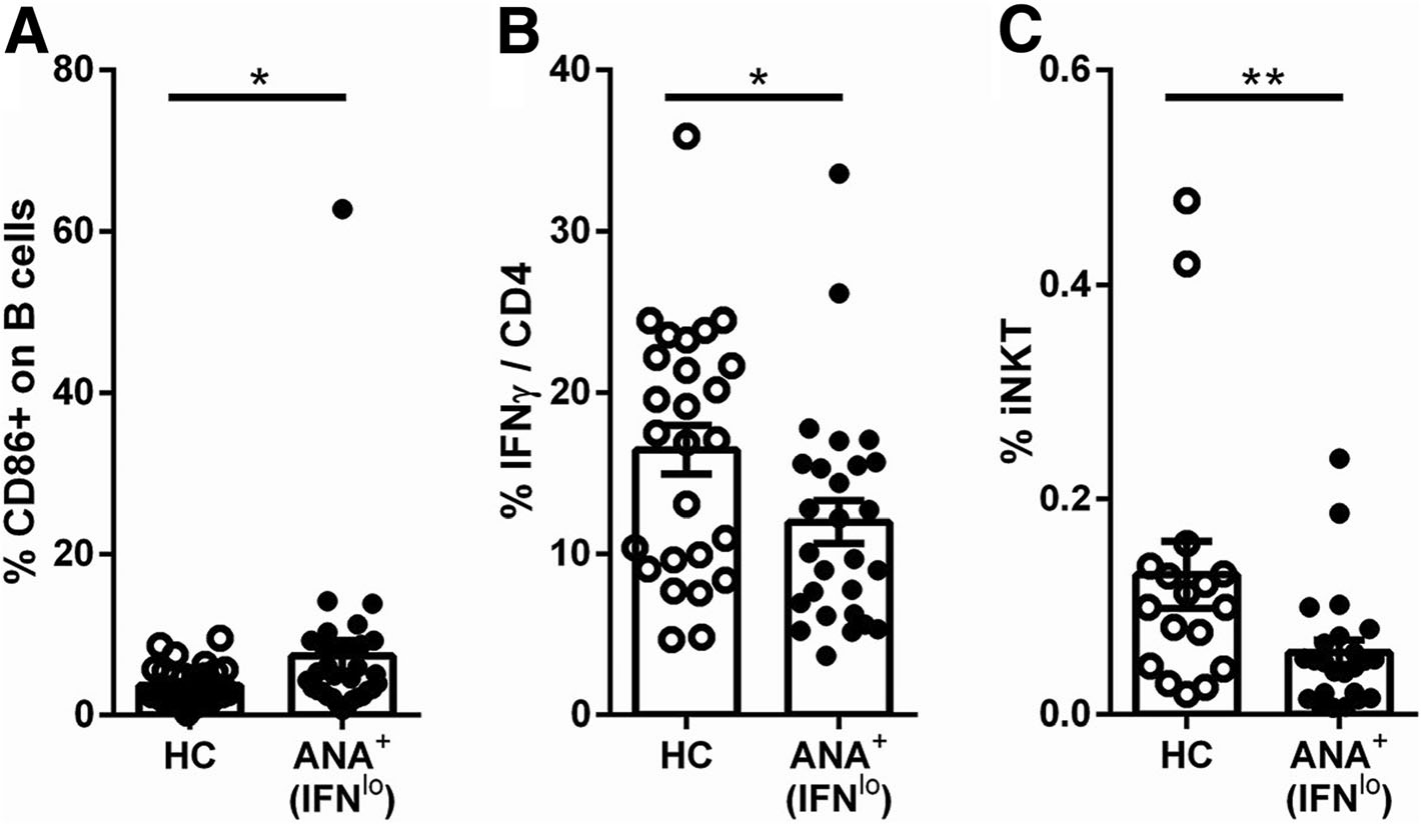
Cellular immunologic changes seen in asymptomatic anti-nuclear antibodies (ANA)^+^ individuals who do not have elevations of type I interferons (IFNs). Asymptomatic ANA^+^ individuals were defined as having normal levels of type I IFNs (IFN^lo^) if their IFN5 score was < 2 SD above the mean for ANA^−^ healthy controls (HC) (all had undetectable levels of serum IFN-α on ELISA). Shown are cellular phenotypes (**a** % CD86^+^ B cells (CD86^+^ on B cells), **b** % CD4 cells producing IFN-γ (IFNγ/CD4) and **c** % invariant natural killer T cells (iNKT))  that were significantly different in IFN^lo^ asymptomatic ANA^+^ individuals as compared to HC. Cell populations were defined and gated as outlined in Figs. 1 and 2. Bars represent the mean with SEM. Every data point represents an individual patient. Statistical significance was determined using the Mann-Whitney *U* test; *p ≤ 0.05, **p ≤ 0.01

Type I IFNs have been previously reported to promote B cell activation, plasma cell differentiation, Tfh and Th1 cell differentiation, and BAFF production [64–69]. Consistent with the possibility that type I IFNs are driving these cellular abnormalities in ANA^+^ individuals, the IFN5 RNA score and/or serum IFN-α levels were positively associated with CD95 +/− CD86 expression on switched memory B cells, PC5 score, percentage of plasma cells, proportion of activated memory Tfh cells, and BAFF RNA and/or serum levels, particularly in the asymptomatic ANA^+^ and early SARD groups (Fig. 3). Nevertheless, a number of cellular abnormalities were still seen in individuals with normal type I IFN levels (IFN5 score <2 SD above mean for ANA^−^ HC), including reduced proportions of IFN-γ-producing CD4^+^ T cells and NKT cells, and increased proportions of CD86^+^ B cells (Fig. 4), indicating that not all of the cellular abnormalities seen in asymptomatic ANA^+^ individuals are induced by elevated type I IFN. Notably, while there was a trend toward increased proportions of activated memory Tfh cells in asymptomatic ANA^+^ individuals with normal levels of type I IFN, this was not statistically significant.

In contrast to type I IFN, elevated proportions of activated memory Tfh cells were not correlated with the majority of cellular phenotypes. However, in asymptomatic ANA^+^ individuals there was a strong positive association (*p* < 0.0001, *r* = 0.70) between the proportion of activated memory Tfh cells and the PC5 score, suggesting a potential involvement of Tfh cells in the differentiation of autoreactive PC and/or plasmablasts in these individuals.

Although we currently have limited follow-up data on the asymptomatic ANA^+^ individuals in our study, 4 out of 28 subjects who were followed for at least 2 years developed SARD symptoms (myositis (*n* = 1), Raynaud’s syndrome (*n* = 1), arthritis (*n* = 1), SLE (*n* = 1)) within the 2 years of follow up. While the majority of phenotypes examined did not differ between progressors and non-progressors, the IFN5 scores and serum IFN-α levels were significantly higher (*p* = 0.023 and 0.048, respectively) and there was a trend toward increased activated memory Tfh cells (*p* = 0.058) in progressors, arguing that these processes may also drive the immune dysregulation leading to progression.

### There is substantial overlap between the immunologic profiles of ANA^+^ individuals with and without symptoms

**Fig. 5:**
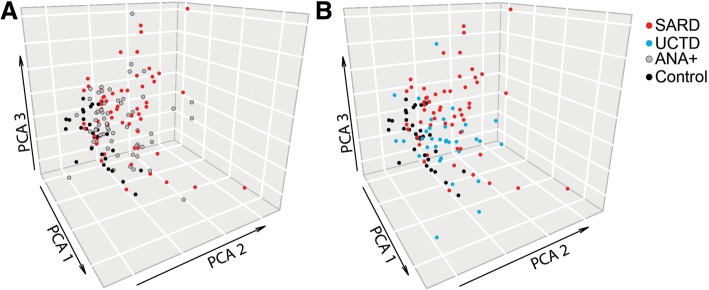
In principal component analysis (PCA) the cellular phenotype of many asymptomatic anti-nuclear antibody (ANA)^+^ individuals or patients with undifferentiated connective tissue disease (UCTD) overlaps with that of patients with systemic autoimmune rheumatic disease (SARD) and is distinct from that of healthy controls. The 3-D plots show asymptomatic ANA^+^ individuals (ANA+, gray) (**a**) or patients with UCTD (blue) **(b**), as compared to healthy controls (black) and patients with SARD (red). Analyses were performed using the PCA function in the missMDA (v1.12) package, with missing data imputed using the imputePCA function. Corresponding plots were created using the scatterplot3d (v0.3–41) package

Since the cellular profiles of ANA^+^ individuals with or without a SARD diagnosis appeared similar on univariate analysis, PCA was performed to determine whether differences between the ANA^+^ groups could be discerned when the data were examined as a whole. As shown in Fig. 5, using 3-dimensional PCA analysis incorporating only cellular immunologic phenotypes and the plasma cell RNA signature, largely independent clusters of patients with SARD and ANA^−^ HC were identified, with most ANA^−^ HC clustered on the lower left in the plots, whereas the majority of the patients with SARD were to the upper right in the plots. While some asymptomatic ANA^+^ subjects and subjects with UCTD appeared to be localized within the region where the majority of ANA^−^ HC were clustered on the PCA plot, a substantial number of these individuals (~ 50%) were admixed with the cluster of patients with early SARD , indicating that their cellular abnormalities were remarkably similar to those seen in patients with early SARD.

## Discussion

In this study, we show that ANA^+^ individuals with no SARD symptoms or insufficient symptoms to make a diagnosis have several cellular immunologic changes in their peripheral blood that are very similar to those seen in newly diagnosed patients who satisfy SARD criteria. These findings indicate that some of the cellular abnormalities that are thought to be characteristic of SARD are actually secondary to the immune dysregulation associated with generation of ANAs rather than the immunologic events that accompany the conversion of asymptomatic to symptomatic autoimmunity.

To our knowledge, this is the first study to comprehensively examine the cellular immune features in asymptomatic ANA^+^ individuals and compare them with patients with UCTD and early SARD. A recent study used flow cytometry to compare PBMCs in asymptomatic ANA^−^ and ANA^+^ healthy individuals, but the number of cell populations examined was limited [70]. This study found a small but significant increase in the proportion of memory (CD27^+^CD38^−^) B cells and plasmablasts (CD27^+^CD38^+^) in ANA^+^ as compared to ANA^−^ individuals, suggestive of chronic B cell activation, but did not examine any activated or specific regulatory T cell populations. We observed a similar trend toward increased plasma cells and plasmablasts in asymptomatic ANA^+^ individuals, but found that the proportion of class-switched memory B cells was reduced, consistent with several previous studies in SARD [28, 30, 33–37]. It is likely that the apparent disparity in memory B cell findings between the two studies results from the differences in the B cell populations examined, as the CD27^+^CD38^−^ memory B cell compartment includes both class-switched and unswitched memory B cell subsets.

There is also a paucity of studies examining the cellular immunologic changes in UCTD. Szordoray et al. found that patients with UCTD and SARD had increased proportions of IFN-γ-producing CD4^+^ T cells and decreased proportions of CD4^+^FOXP3^+^CD25^+^ T cells as compared to healthy controls [54]. In a subsequent study, the same group reported an increased ratio of IL-17-producing CD4^+^ T cells to Treg cells in patients with UCTD, with further increases in patients progressing to SARD [71]. Our findings contrast with these results, showing a trend toward decreased proportions of IFN-γ-producing CD4^+^ T cells and increased proportions of CD4^+^FOXP3^+^HELIOS^+^ Treg cells in UCTD, both of which were statistically significant in SARD. Currently, the explanation for these differences is unclear. While there were some minor differences between the two studies in the stains used to identify the T cell populations and in how the data were expressed, analysis of our data using comparable gating still yielded discordant results. Of particular note, although there was a non-significant trend toward a decreased proportion of CD25^+^ cells within the CD4^+^FOXP3^+^HELIOS^+^ Treg cell subset in early SARD (ANA^−^ HC 88.37, asymptomatic ANA^+^ individuals 86.17, patients with UCTD 86.12, patients with SARD 83.53, all *p* > 0.05), the proportion of CD4^+^FOXP3^+^HELIOS^+^CD25^+^ cells remained elevated as compared to ANA^−^ HC, with similar trends seen in asymptomatic ANA^+^ patients (*p* < 0.05) and patients with UCTD.

Our results showing significant increases in the proportion of CD4^+^FOXP3^+^HELIOS^+^ Treg cells in asymptomatic ANA^+^ individuals and patients with early SARD recapitulate recent findings in SLE and SjD [45, 47, 48], and collectively suggest that ongoing activation of T cell subsets in ANA^+^ individuals outstrips the capacity of Treg cells to regulate them. These findings are at odds with previous studies suggesting that progression from asymptomatic ANA positivity or UCTD to SARD results from impaired immunoregulation [54, 70, 71]. Indeed, we found that the ratio of cytokine-producing or Tfh cells to Treg cells did not differ between any of the ANA^+^ groups and that the proportion of Treg cells was moderately correlated with the proportion of Tfh cells in all ANA^+^ groups, suggesting similar attempts at immunoregulation. It is currently unknown whether the expanded Treg populations in any of the ANA^+^ groups are functionally altered.

The nature of the immunologic changes seen in asymptomatic ANA^+^ individuals suggests that abnormal activation of both T and B cells contributes to the generation of ANAs. While some of these immunologic changes may be driven by the elevated type I IFN seen in a subset of these individuals [27], increased B cell activation and decreased proportions of iNKT and IFN-γ-producing CD4^+^ T cells were still seen in asymptomatic ANA^+^ individuals with type I IFN within the normal range. Although we have not previously assessed cytokine-producing T cells in asymptomatic ANA^+^ individuals, the iNKT findings observed in the current study recapitulate those seen in our previous study of asymptomatic first-degree relatives of patients with SLE, where we found an association between reduced levels of iNKT cells and ANA positivity [44]. In that study, we found that the levels of iNKT cells were significantly correlated between genetically related individuals in the same family suggesting that this was a heritable trait. Given the evidence that iNKT cells are involved in regulation of autoimmunity [72], these findings suggest that deficiencies of iNKT cells may predispose genetically susceptible individuals to the development of ANA positivity.

It is probable that the decreased proportion of peripheral blood IFN-γ-producing CD4^+^ T cells observed in our study reflects chronic activation, which could arise either from migration into inflamed tissues [61] or exhaustion due to chronic activation [73]. Since altered activation of IFN-γ-producing CD4^+^ T cells is seen in asymptomatic ANA^+^ individuals with normal levels of type I IFN and since we and others have found that elevated type I IFN typically follows autoantibody production, this observation suggests that activation of IFN-γ-producing cells temporally precedes type I IFN production in the evolution of ANAs. This concept has previously been proposed based upon the observation that elevated serum IFN-γ temporally precedes increases in type I IFNs during progression to SLE [74].

The proportion of Tfh cells was also increased in asymptomatic ANA^+^ individuals. In this study, we gated these cells as CXCR5^+^PD1^hi^, which in previous work have been shown to represent activated memory Tfh cells that are released into the circulation following germinal center-inducing immune responses [49, 75]. Although activated memory Tfh have been shown to secrete IL-21, no differences in the proportion of IL-21-secreting CD4^+^ T cells were observed between groups, likely because activated memory Tfh only represent a small proportion of IL-21-secreting cells in the peripheral blood [76], precluding detection of significant differences.

As outlined previously, in asymptomatic ANA^+^ individuals, the proportion of Tfh strongly correlated with PC5 score, suggesting a potential involvement of Tfh in autoantibody production. Consistent with this possibility, the proportion of Tfh was positively correlated with the ANA titer in asymptomatic ANA^+^ individuals and with the number of autoantibody specificities in patients with SARD, and was also an independent predictor of serologic status in multivariate analysis including all ANA^+^ subjects. These findings are compatible with previous work in mouse models indicating that the T-B collaboration within germinal centers is important in epitope spreading and production of high-affinity pathogenic autoantibodies [77, 78]. Although type I IFN levels were also independently associated with the number of autoantibody specificities in the multivariate analysis, it is likely that they too act through this process, as type I IFNs have been shown to have a number of immunologic effects that enhance Tfh development and germinal center responses [65, 67, 68]. Notably, asymptomatic ANA^+^ individuals who developed new SARD criteria over the subsequent 2 years had significantly higher type I IFN and a trend toward increased proportions of Tfh cells, suggesting that these processes also drive progression.

In many ANA^+^ patients and patients with UCTD the cellular phenotypes examined could not be discriminated from those of patients with early SARD. This finding suggests that there are additional as yet undetermined immunologic events that dictate the onset of symptoms in SARD. Previous work has shown that the onset of symptoms in SARD is associated with increasing production of pro-inflammatory factors [79]; thus, there must be a change in the character of the immune response that results in the development of symptoms. Whether this is due to alterations in the amount, specificity, or character of autoantibodies, availability of autoantigens, nature of T cell help, or immunoregulatory function is currently unknown and is the focus of ongoing investigations.

## Conclusions

Asymptomatic ANA^+^ individuals and patients with UCTD have abnormal activation of their peripheral blood B and T cell compartments. The types of immunologic changes seen suggest that germinal centers are important in the generation of ANAs and that type I IFNs may enhance this process. Given the similarity between the immune abnormalities in these individuals and those with early SARD, some of the currently accepted cellular features of SARD appear to be associated with ANA production rather than the immunologic events that cause symptoms in SARD.

## Additional file

Additional file 1: **Table S1.** Study participant characteristics. **Figure S1.** Proportion of CD95^+^ cells in the peripheral B cell subsets of ANA^+^ individuals with and without SARD. **Figure S2.** Plasma cell and plasmablast frequencies are unchanged in ANA^+^ individuals with or without a SARD diagnosis. **Figure S3.** The majority of cellular phenotypes that differ between ANA^+^ and ANA^-^ groups do not vary with age. **Figure S4.** BAFF and type I IFN levels are increased in SARD patients. **Figure S5.** Spearman correlation matrix showing the association between cellular and selected serologic/cytokine phenotypes in UCTD patients. (DOCX 1697 kb)

## Abbreviations

ANA: Anti-nuclear antibody
BAFF: B cell activating factor
ELISA: Enzyme-linked immunosorbent assay
HC: Healthy control
IFN: Interferon
IL: Interleukin
iNKT: Invariant natural killer T
PBMC: Peripheral blood mononuclear cell
PC: Plasma cell
PCA: Principal component analysis
PMA: Phorbol-12-myristate-13-acetate
SARD: Systemic autoimmune rheumatic disease
SjD: Sjogren’s Disease
SLE: Systemic lupus erythematosus
SSc: Systemic sclerosis
Tfh: T follicular helper
Treg: T regulatory
UCTD: Undifferentiated connective tissue disease
