# Supplementary material for: The presence of anti-nuclear antibodies alone is associated with changes in B cell activation and T follicular helper cells similar to those in systemic autoimmune rheumatic disease
Source: Arthritis Res Ther. 2018 Nov 29;20:264. doi: 10.1186/s13075-018-1752-3 (PMC6263058; doi:10.1186/s13075-018-1752-3)
Supplement: Supplementary file 1 — Table S1. Study participant characteristics. Figure S1. Proportion of CD95+ cells in the peripheral B cell subsets of ANA+ individuals with and without SARD. Figure S2. Plasma cell and plasmablast frequencies are unchanged in ANA+ individuals with or without a SARD diagnosis. Figure S3. The majority of cellular phenotypes that differ between ANA+ and ANA- groups do not vary with age. Figure S4. BAFF and type I IFN levels are increased in SARD patients. Figure S5. Spearman correlation matrix showing the association between cellular and selected serologic/cytokine phenotypes in UCTD patients. (DOCX 1697 kb) [file 13075_2018_1752_MOESM1_ESM.docx]

**Table S1.** Study participant characteristics.

|  | **ANA^-^ Healthy Control**  **N=32** | **ANA^+^**  **No Symps**  **N=61** | **UCTD**  **N=35** | **SARD**  **N=59** | **SSc**  **N=19** | **SLE**  **N=10** | **SjD**  **N=28** | **DM/MCTD**  **N=2** |
| --- | --- | --- | --- | --- | --- | --- | --- | --- |
| **Ethnicity:** Non-Caucasian N (%)  African American  South East Asian  Asian  Hispanic  Filipino  Mixed | 20 (62.5)  1 (3.1)  7 (21.9)  1 (3.1)  7 (21.9)  1 (3.1)  3 (9.4) | 25 (41.0)  7 (11.5)  6 (9.8)  5 (8.2)  2 (3.3)  1 (1.6)  4 (6.6) | 11 (31.4)  1 (2.9)  3 (8.6)  5 (14.3)  1 (2.9)  0 (0)  1 (2.9) | 20 (33.9)  1 (1.7)  6 (10.2)  4 (6.8)  4 (6.8)  2 (3.4)  3 (5.1) | 6 (31.6)  0 (0)  2 (10.5)  1 (5.3)  3 (15.8)  0 (0)  0 (0) | 5 (50)  1 (10)  1 (10)  0 (0)  1 (10)  1 (10)  1 (10) | 8 (28.6)  0 (0)  3 (10.7)  3 (10.7)  0 (0)  0 (0)  2 (7.1) | 1 (50)  0 (0)  0 (0)  0 (0)  0 (0)  1 (50)  0 (0) |
| **Specific Abs:** N (%)  dsDNA  Ro  La  Sm  Sm/RNP  RNP  Scl-70  Centromere  Chromatin | 0 (0)  0 (0)  0 (0)  0 (0)  0 (0)  0 (0)  0 (0)  0 (0)  0 (0) | 4 (6.6)  15 (24.6)  7 (11.5)  2 (3.3)  3 (4.9)  7 (11.5)  1 (1.6)  1 (1.6)  5 (8.2) | 3 (8.6)  10 (28.6)  2 (5.7)  2 (5.7)  4 (11.4)  5 (14.3)  1 (2.9)  3 (8.6)  3 (8.6) | 8 (13.6)  35 (59.3)  21 (35.6)  4 (6.8)  6 (10.2)  8 (13.6)  9 (15.3)  16 (27.1)  7 (11.9) | 2 (10.5)  3 (15.8)  0 (0)  0 (0)  1 (5.3)  1 (5.3)  5 (26.3)  13 (68.4)  0 (0) | 3 (30)  4 (40)  1 (10)  3 (30)  4 (40)  4 (40)  2 (20)  1 (10)  5 (50) | 3 (10.7)  28 (100)  20 (71.4)  0 (0)  0 (0)  2 (7.1)  2 (7.1)  1 (3.6)  1 (3.6) | 0 (0)  0 (0)  0 (0)  1 (50)  1 (50)  1 (50)  0 (0)  1 (50)  1 (50) |
| **No. Specific Abs:** N (%)  0  1  2  3  4  ≥5 | 0 (0)  0 (0)  0 (0)  0 (0)  0 (0)  0 (0) | 33 (54.1)  18 (29.5)  6 (9.8)  2 (3.3)  1 (1.6)  1 (1.6) | 13 (37.1)  17 (48.6)  3 (8.6)  0 (0)  0 (0)  2 (5.7) | 3 (5.1)  21 (35.6)  25 (42.4)  4 (6.8)  4 (6.8)  2 (3.4) | 1 (5.3)  13 (68.4)  4 (21.1)  0 (0)  1 (5.3)  0 (0) | 2 (20)  2 (20)  2 (20)  1 (10)  1 (10)  2 (20) | 0 (0)  4 (14.3)  20 (71.4)  3 (10.7)  1 (3.6)  0 (0) | 0 (0)  1 (50)  0 (0)  0 (0)  1 (50)  0 (0) |
| **ANA Titer:** N (%)  1/160  1/320  1/640  >1/640 | 0 (0)  0 (0)  0 (0)  0 (0) | 18 (29.5)  7 (11.5)  17 (27.9)  19 (31.1) | 7 (20)  7 (20)  15 (42.9)  6 (17.1) | 2 (3.4)  8 (13.6)  14 (23.7)  35 (59.3) | 0 (0)  0 (0)  2 (10.5)  17 (89.5) | 0 (0)  3 (30)  2 (20)  5 (50) | 2 (7.1)  5 (17.9)  10 (35.7)  11 (39.3) | 0 (0)  0 (0)  0 (0)  2 (100) |

Abbreviations: N, number; SD, standard deviation; Abs, antibodies

*All patients that were anti-La Ab positive were anti-Ro Ab positive, except for 1 patient with UCTD


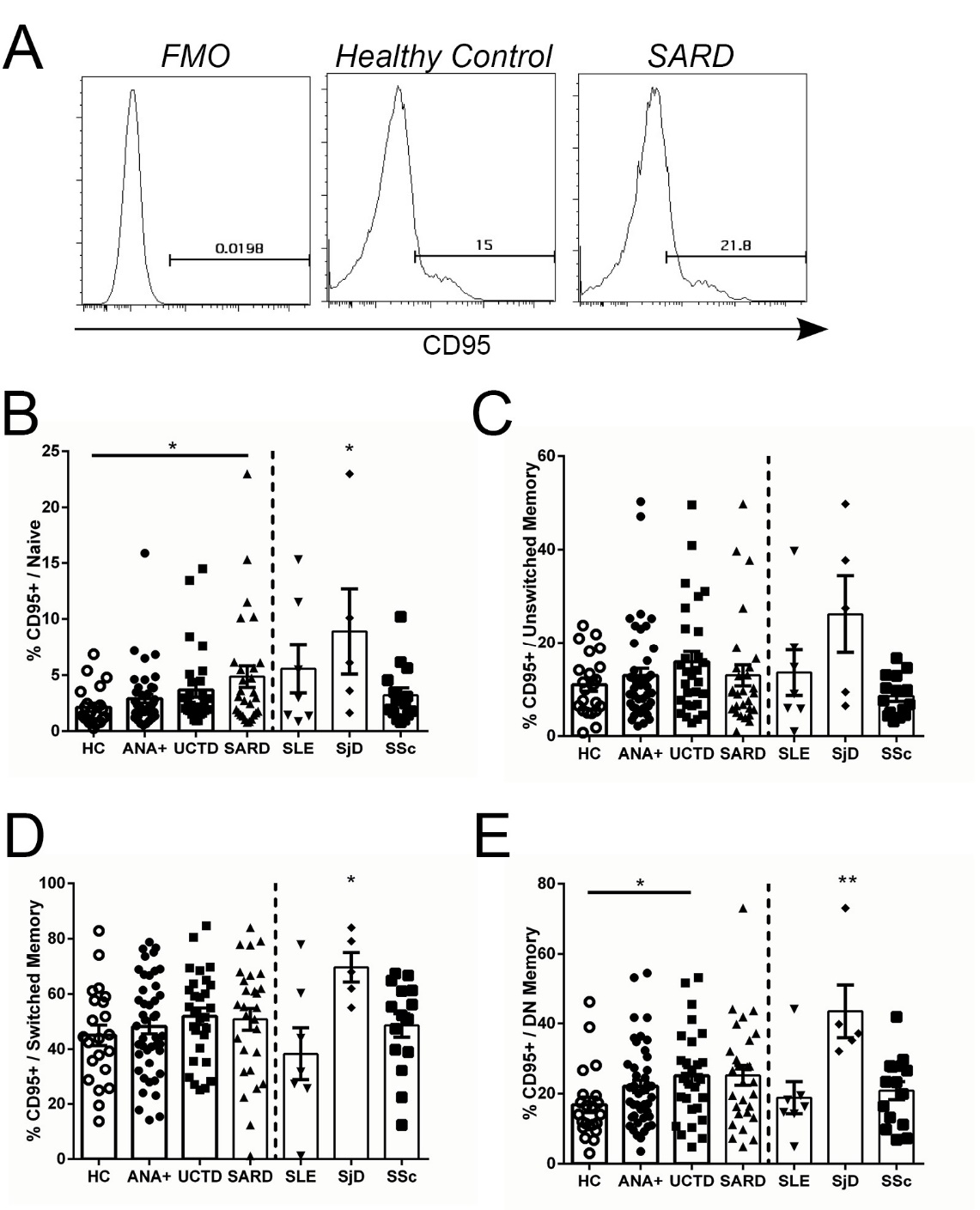


**Figure S1: Proportion of CD95^+^ cells in the peripheral B cell subsets of ANA^+^ individuals with and without SARD.** **(A)** Representative gating for CD95^+^ B cells (gated on CD19^+^ cells). **(B-E)** Scatterplots showing the proportion of CD95^+^ cells in the different peripheral B cell compartments. Statistical comparisons on the left side of each figure are between healthy controls (HC) and asymptomatic ANA^+^ (ANA+), UCTD, or pooled SARD patients, whereas those on the right side of the figure are between the individual SARDs and HC. Bars represent the mean with SEM. Every data point represents an individual patient. For each set of comparisons statistical significance was determined using the Kruskal-Wallis test with Dunn’s post-test for multiple comparisons, as compared to HC. * p ≤ 0.05, ** p ≤ 0.01.


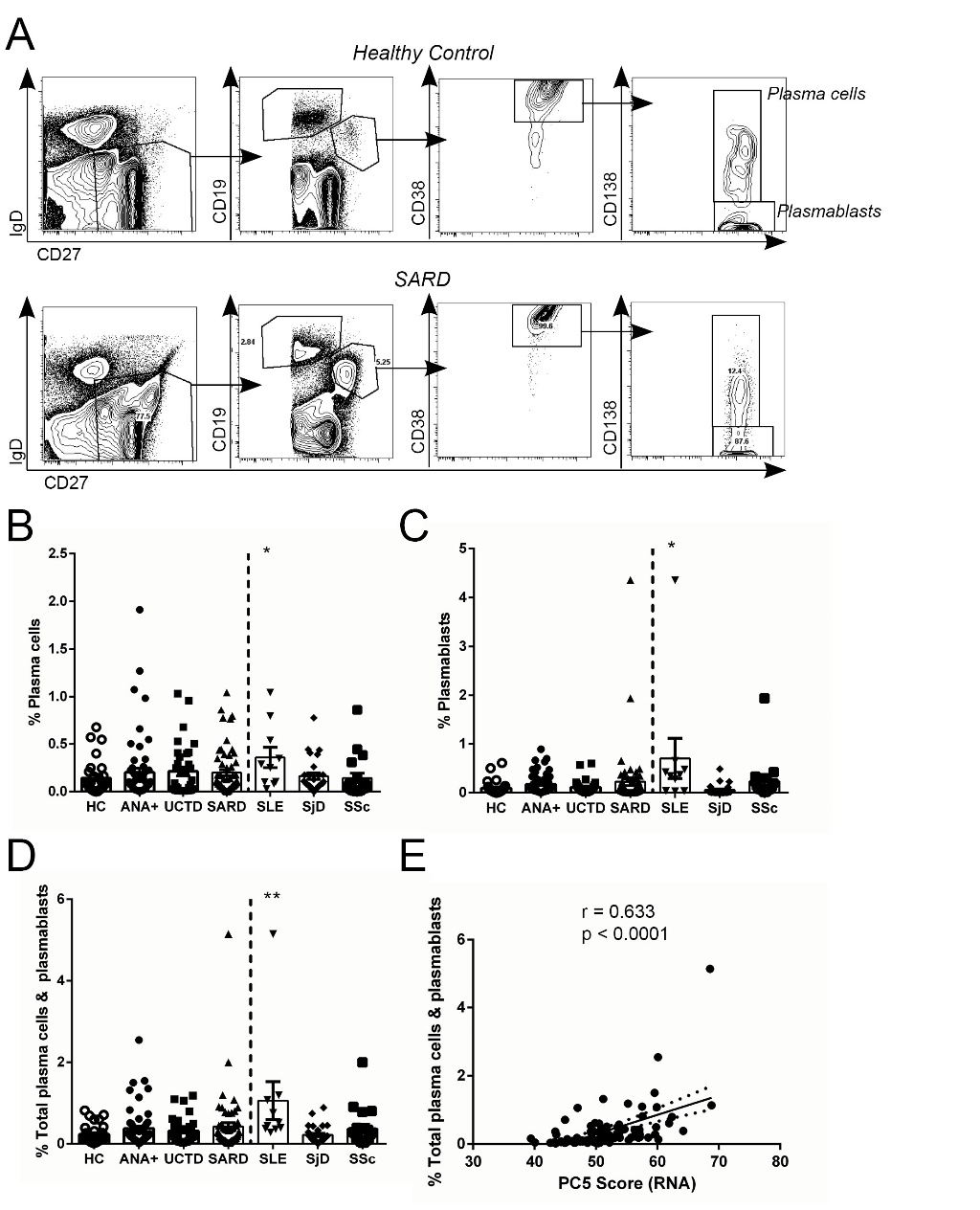


**Figure S2: Plasma cell and plasmablast frequencies are unchanged in ANA^+^ individuals with or without a SARD diagnosis. (A)** Representative gating for plasma cells and plasmablasts in PBMCs. **(B-D)** Scatterplots showing the percentage of **(B)** plasma cells, **(C)** plasmablasts, and **(D)** combined total plasma cells/plasmablasts in the peripheral blood for each of the subject groups. **(E)** Correlation between the PC5 score (RNA) and total plasma cell/plasmablast frequency in the peripheral blood. Statistical comparisons on the left side of each figure are between healthy controls (HC) and asymptomatic ANA^+^ (ANA+), UCTD, or pooled SARD patients, whereas those on the right side of the figure are between the individual SARDs and HC. Bars represent the mean with SEM. Every data point represents an individual patient. For each set of comparisons statistical significance was determined using the Kruskal-Wallis test with Dunn’s post-test for multiple comparisons, as compared to HC. * p ≤ 0.05, ** p ≤ 0.01, ***p ≤ 0.001, **** p ≤ 0.0001.

A

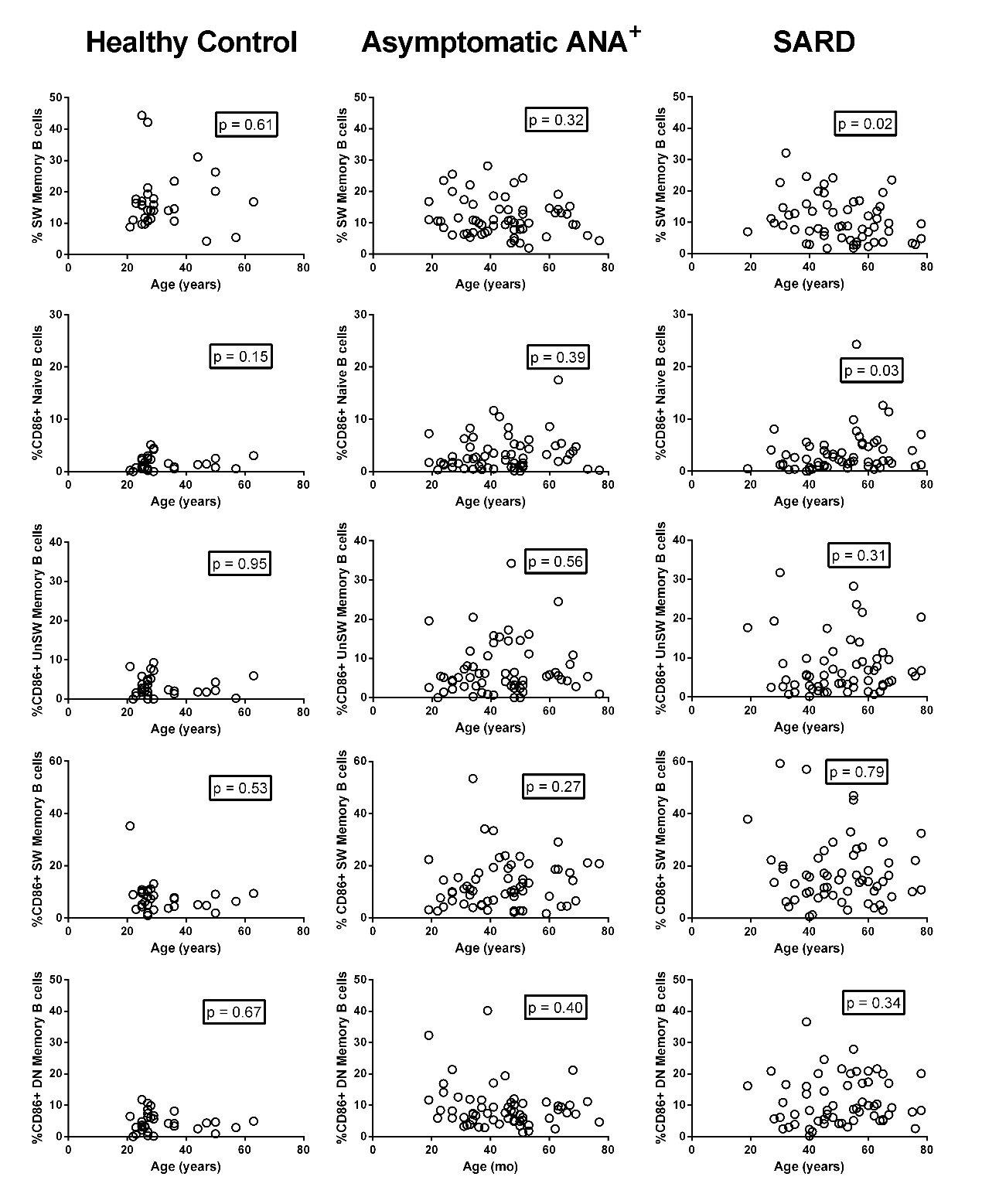


B


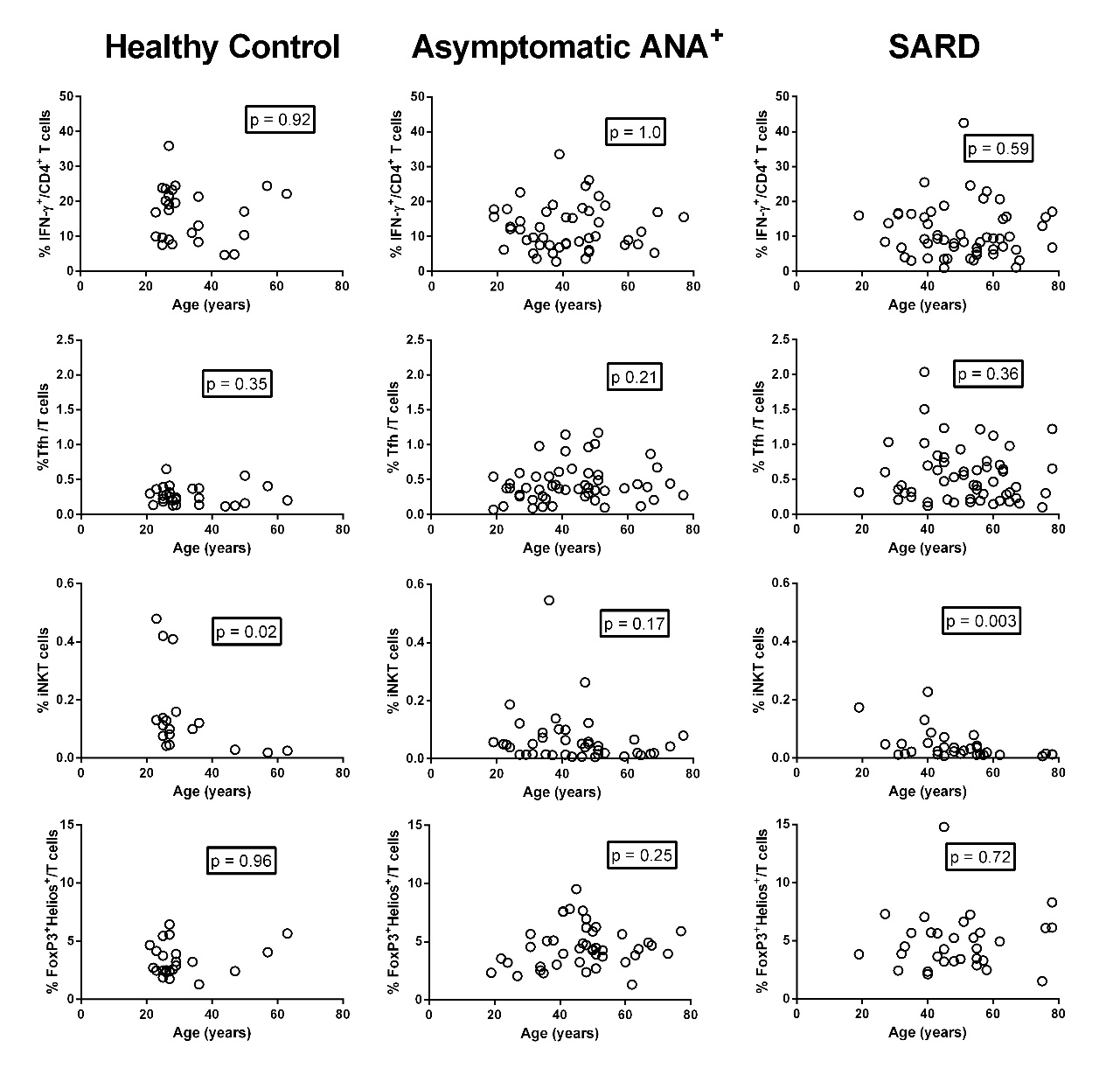


**Figure S3: The majority of cellular phenotypes that differ between ANA^+^ and ANA^-^ groups do not vary with age.** Plots showing the correlation between age (years) and various B **(A)** and T **(B)** cell phenotypes (defined and gated as outlined in Figures 1 and 2). The p-values for the significance of the association are shown in the top right corner of each graph and were calculated using the Spearman correlation coefficient.


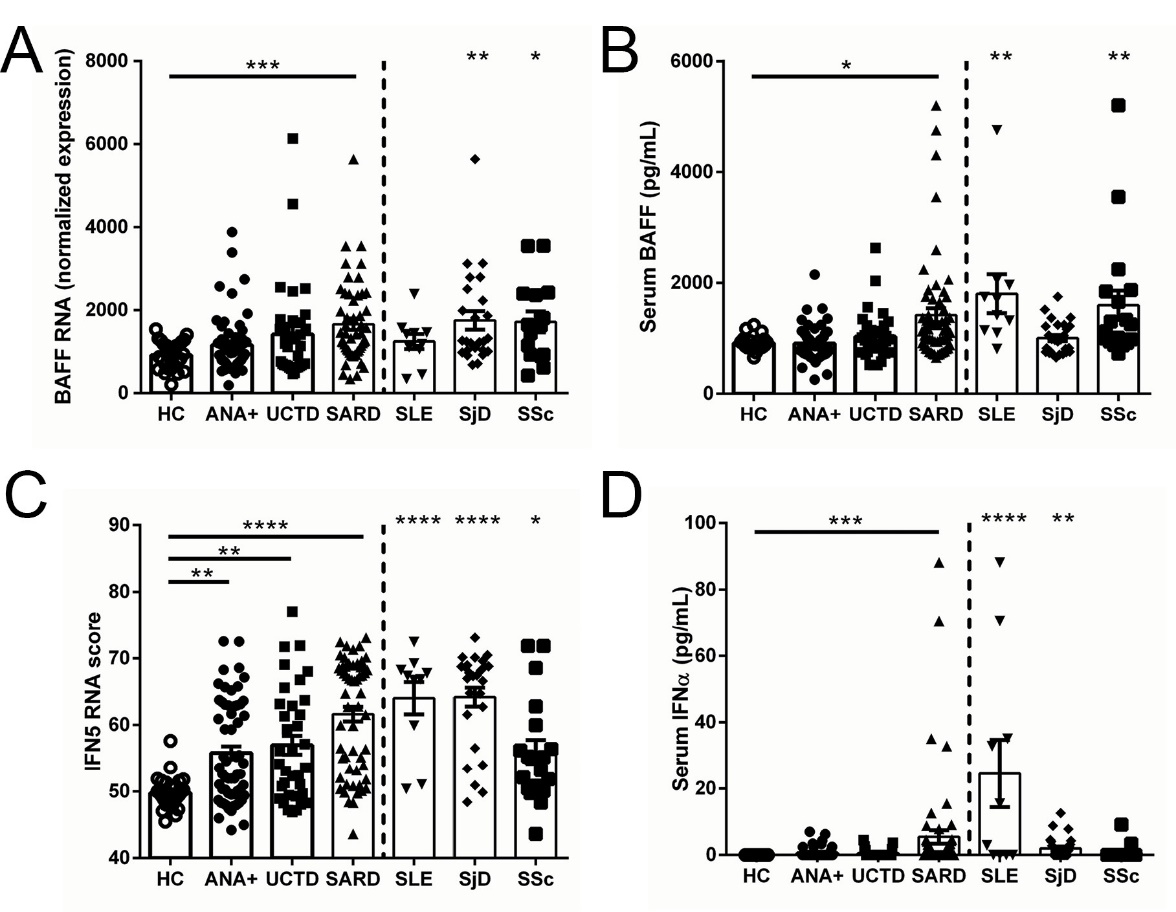


**Figure S4: BAFF and type I IFN levels are increased in SARD patients.** Scatterplots showing: **(A)** normalized peripheral blood BAFF RNA expression; **(B)** Serum BAFF levels; **(C)** Peripheral blood IFN5 score; and **(D)** serum IFN-α levels, for each of the subject groups. Statistical comparisons on the left side of each figure are between healthy controls (HC) and asymptomatic ANA^+^ (ANA+), UCTD, or pooled SARD patients, whereas those on the right side of the figure are between the individual SARDs and HC. Bars represent the mean with SEM. Every data point represents an individual patient. For each set of comparisons statistical significance was determined using the Kruskal-Wallis test with Dunn’s post-test for multiple comparisons, as compared to HC. * p ≤ 0.05, ** p ≤ 0.01, ***p ≤ 0.001, **** p ≤ 0.0001. Some of the data has been previously published in [26].

**
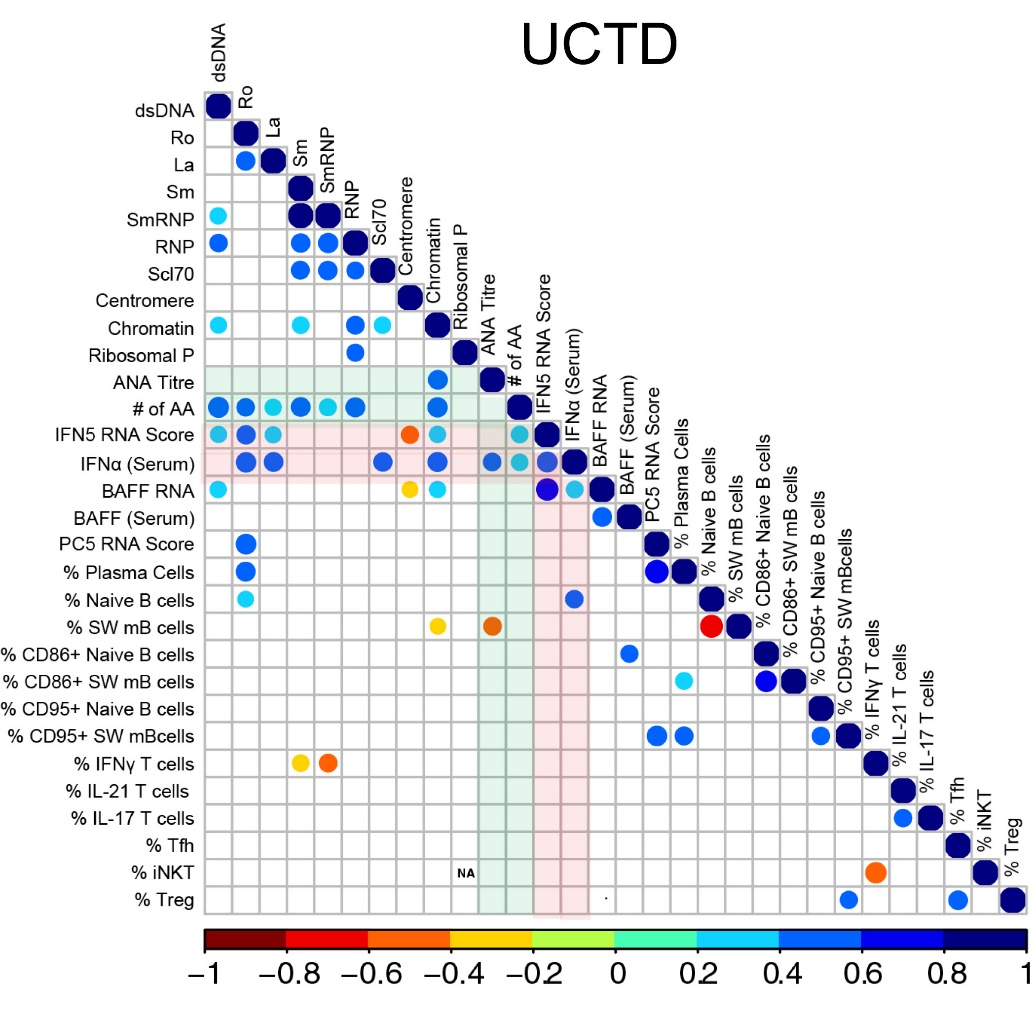
**

**Figure S5. Spearman correlation matrix showing the association between cellular and selected serologic/cytokine phenotypes in UCTD patients.** B and T cell populations were gated and defined as outlined in Figures 1 and 2, plasma cells as outlined in Figure S3, and CD95^+^ B cell subpopulations as shown in Figure S1. The color and size of the dots represents the ρ value, with the scales shown at the bottom of each matrix. Non-significant (p ≥ 0.05) correlations are not displayed.
